# Pertussis Post-Exposure Prophylaxis among Household Contacts: A Cost-Utility Analysis

**DOI:** 10.1371/journal.pone.0119271

**Published:** 2015-03-06

**Authors:** Nisha Thampi, Ipek Gurol-Urganci, Natasha S. Crowcroft, Beate Sander

**Affiliations:** 1 Division of Infectious Diseases, Children’s Hospital of Eastern Ontario, Ottawa, ON, Canada; 2 University of Ottawa, Ottawa, ON, Canada; 3 Department of Health Services Research and Policy, London School of Hygiene and Tropical Medicine, London, United Kingdom; 4 Laboratory Medicine and Pathobiology, University of Toronto, Toronto, ON, Canada; 5 Public Health Ontario, Toronto, ON, Canada; 6 Institute of Health Policy, Management & Evaluation, University of Toronto, Toronto, ON, Canada; 7 Institute for Clinical Evaluative Sciences, Toronto, ON, Canada; 8 Toronto Health Economics and Technology Assessment Collaborative, Toronto, ON, Canada; Glaxo Smith Kline, DENMARK

## Abstract

**Background:**

Recent pertussis outbreaks have prompted re-examination of post-exposure prophylaxis (PEP) strategies, when immunization is not immediately protective. Chemoprophylaxis is recommended to household contacts; however there are concerns of clinical failure and significant adverse events, especially with erythromycin among infants who have the highest disease burden. Newer macrolides offer fewer side effects at higher drug costs. We sought to determine the cost-effectiveness of PEP strategies from the health care payer perspective.

**Methods:**

A Markov model was constructed to examine 4 mutually exclusive strategies: erythromycin, azithromycin, clarithromycin, or no intervention, stratified by age group of contacts (“infant”, “child”, and “adult”). Transition probabilities, costs and quality-adjusted life years (QALYs) were derived from the literature. Chronic neurologic sequelae were modeled over a lifetime, with costs and QALYs discounted at 5%. Associated health outcomes and costs were compared, and incremental cost-effectiveness ratios (ICER) were calculated in 2012 Canadian dollars. Deterministic and probabilistic sensitivity analyses were performed to evaluate the degree of uncertainty in the results.

**Findings:**

Azithromycin offered the highest QALYs in all scenarios. While this was the dominant strategy among infants, it produced an ICER of $16,963 per QALY among children and $2,415 per QALY among adults. Total QALYs with azithromycin were 19.7 for a 5-kg infant, 19.4 for a 10-year-old child, and 18.8 for a 30-year-old adult. The costs of azithromycin PEP among infants, children and adults were $1,976, $132 and $90, respectively. While results were sensitive to changes in PEP effectiveness (11% to 87%), disease transmission (variable among age groups) and hospitalization costs ($379 to $59,644), the choice of strategy remained unchanged.

**Interpretation:**

Pertussis PEP is a cost-effective strategy compared with no intervention and plays an important role in contact management, potentially in outbreak situations. From a healthcare payer perspective, azithromycin is the optimal strategy among all contact groups.

## Introduction

In the pre-vaccine era, pertussis was a major childhood illness affecting children primarily under 10 years of age, and a major cause of death among infants under 1 year [1]. Caused by *Bordetella pertussis*, the disease was pandemic throughout the 20^th^ century, with cyclical epidemic peaks every 2 to 5 years [1]. Widespread immunization of children has not lengthened the epidemic cycle as much as expected [2], suggesting that adults serve as a reservoir for disease in young children [3]; pertussis is common and endemic among this group [4,5].

Prolonged pertussis outbreaks have recently been reported across North America [6–9], prompting re-examination of control and prevention strategies [10]. In 2010, 101 cases of pertussis were reported in Ontario (0.8 cases per 100,000 population) [6]. Following a 2011–2012 outbreak, 792 cases were reported across both years, or 5.9 per 100,000 population. Immunization coverage in Ontario among 7- and 17-year olds in 2012 was 76% and 68%, respectively [6].

The resurgence of pertussis has been attributed to waning immunity in older children and poor vaccine efficacy against other *Bordetella* species associated with pertussis-like illnesses [7,11,12]. A randomized, controlled trial (RCT) of post-exposure prophylaxis (PEP) with erythromycin compared with placebo found PEP to be effective in preventing secondary cases in 67.5% of adult and pediatric household contacts [13]. Practice guidelines in Canada, the United States and the United Kingdom recommend chemoprophylaxis with a macrolide for household and close contacts of pertussis cases of all ages, irrespective of immunization status [14–16]. While studies have examined erythromycin as a treatment and PEP agent, the newer macrolides, azithromycin and clarithromycin, have also been shown to be active against *B*. *pertussis* in vitro [17], and have been recommended as better-tolerated, equally effective [18,19] but more costly treatment options [15,16].

Economic evaluations can provide “value-for-money” information to support health care decision-making [20]. A cost-utility analysis was performed to compare the additional cost per QALY gained across the 4 strategies of erythromycin, azithromycin, clarithromycin and no prophylaxis, with an aim to estimate the effects, costs and cost-effectiveness of alternative strategies for the prevention of pertussis among household contacts. This analysis is especially timely in informing clinical practice and health care resource allocation given the recent outbreaks among under-immunized populations in countries that have well-established immunization programs.

## Methods

### Cost-utility analysis

In accordance with established guidelines for economic evaluations [20,21], a cost-utility analysis was performed to determine whether PEP with a macrolide was cost-effective compared to no PEP (i.e. treatment of cases as they arise) in household contacts exposed to pertussis. Specific PEP regimens considered were erythromycin and azithromycin in all age groups (infants, children and adults) and clarithromycin in children and adults only. The primary outcome was the incremental cost-effectiveness ratio (ICER), derived from estimated quality-adjusted life years (QALYs) and direct healthcare cost over a lifetime time horizon. The analysis was conducted from the healthcare payer perspective (i.e. Ontario’s Ministry of Health and Long-Term Care), with a commonly-used cost-effectiveness threshold of $50,000 per QALY gained [22]. Healthcare costs for PEP and the treatment of cases are included. All costs were expressed in 2012 Canadian dollars (1 US dollar was 0.99958 Canadian dollars) [23–26]. Costs and health outcomes were discounted at 5% per year as recommended for health economic evaluations in Canada [20].

### Model and assumptions

A Markov model was constructed to evaluate the lifetime health outcomes and costs of developing pertussis among household contacts following expectant management or PEP. Most events occurred in the first year. The Markov model had a cycle length of 1 year and incorporated 2 health states: survival and death. The duration of acute events in the first year was taken into account. Household contacts were stratified into 3 age groups: less than 1 year of age (“infant”), 1 to 12 years old (“child”) and greater than 12 years old (“adult”).

**Fig 1:**
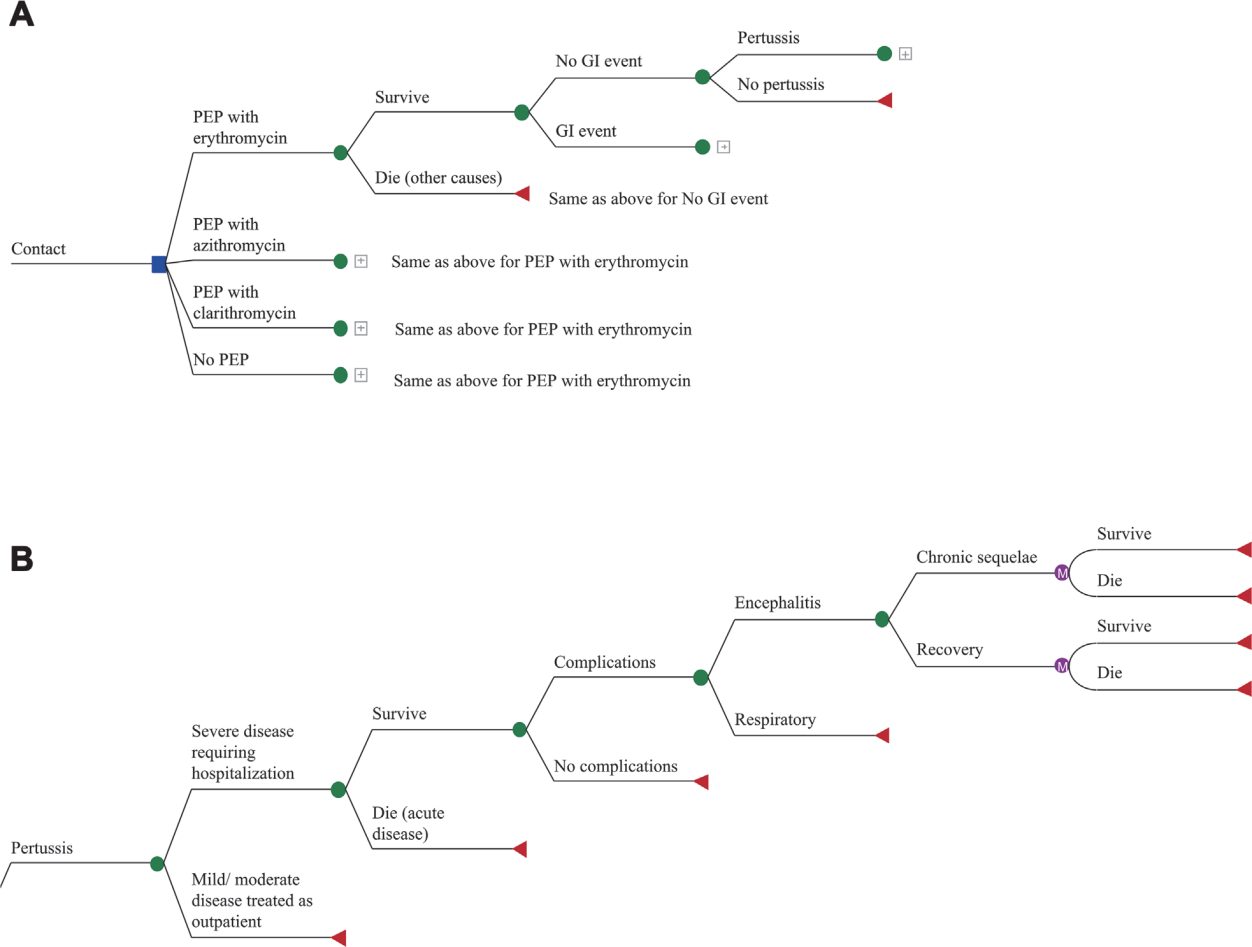
Markov model for post-exposure prophylaxis strategy. The square represents a decision node, and circle represents a chance node. The triangle represents the final outcome for that event pathway. Consequences associated with the chance node are mutually exclusive. PEP: post-exposure prophylaxis; GI: gastrointestinal.

The model structure was the same for all contacts (Fig. 1A and B). Infant contacts had a higher frequency of hospitalization for severe disease, including significant respiratory or neurologic complications. Children and adult contacts with respiratory symptoms were managed in the outpatient setting depending on severity, although all patients with neurologic presentations required hospitalization. Household contacts that developed mild symptoms went to their general practitioner (GP) rather than an emergency department. Mild-to-moderate cough resulted in an additional GP visit and antibiotics, and those with severe cough or pneumonia had 2 additional GP visits, antibiotics and a chest x-ray. Survivors of respiratory disease requiring hospitalization did not experience long-term sequelae. In all groups, pertussis-related deaths were assumed to occur during hospitalization. Age-specific mortality was obtained from Statistics Canada [27]. Given that the total duration of illness is 6–8 weeks [1], all costs attributable to pertussis were assumed to have been incurred in the first year unless patients experienced neurological sequelae, which were assumed to require lifetime healthcare services.

### Data

Probabilities of disease-specific outcomes and medication-related gastrointestinal effects were drawn from RCTs and observational cohort studies. All data are shown in Table 1. Secondary attack rates of pertussis are highest among young infants at 65% [28], with a 10-fold decrease among children and adults [13]. Similarly, more than two-thirds of infants with pertussis are managed in hospital [29], compared to only 7% of children [30] and 3.5% of adult contacts [29]. Encephalitis is an infrequent but serious complication of pertussis, affecting 0.5% infants [31], 0.08% children [30] and 0.05% adults who are hospitalized [30]. Early economic evaluations and population studies reported residual effects in one-third of survivors of pertussis encephalitis [1,32,33]. Death is least likely to occur among adult patients with pertussis, at 0.01%, followed by children at 0.06%. The probability of death is highest among infants, at 0.6% [29].

**Table 1:** Event probabilities for decision model.

**Contact group**	**Parameter**	**Base case value**	**Range examined**	**References**
ALL	Prophylaxis effectiveness	0.675	0.076–0.887	[13]
INFANT				
Intervention	IHPS following erythromycin	0.0128	0.0026–0.0275	[35]
	IHPS following azithromycin	0.0005	0.0001–0.0010	Assumption^1^
Pertussis	Develops pertussis post-exposure	0.65	0.25–0.81	[28,70]
	Hospitalization for severe disease	0.69	0.59–0.82	[29,30]
	Develops complication in hospital	0.14	0.10–0.19	[42,66]
	Death	0.006	0.001–0.009	[29,66,82]
	Develops encephalitis	0.005	0.002–0.009	[29,31,42]
	Develops chronic neurologic sequelae	0.33	0.25–0.50	[32,33]
CHILD				
Intervention	GI adverse event with erythromycin	0.34	0.27–0.44	[13,19,28]
	GI adverse event with azithromycin	0.19	0.12–0.20	[18]
	GI adverse event with clarithromycin	0.32	0.20–0.40^2^	[19]
Pertussis	Acquires pertussis post-exposure	0.061	0.048–0.440	[13,28]
	Develops moderate to severe respiratory pertussis	0.25	0.10–0.32	[42,83]
	Hospitalization for severe disease	0.07	0.04–0.08	[29,30,58]
	Death	0.0006	0.0004–0.0010	[29,30,70]
	Develops encephalitis/ encephalopathy	0.0008	0.0005–0.0040	[29,30,32]
	Develops chronic neurologic sequelae	0.33	0.25–0.50	[1,32,33]
ADULT				
Intervention	GI adverse event with erythromycin	0.34	0.27–0.44	[13,19,28]
	GI adverse event with azithromycin	0.19	0.12–0.20	[18]
	GI adverse event with clarithromycin	0.32	0.2–0.40^2^	[19]
Pertussis	Acquires pertussis post-exposure	0.061	0.048–0.200	[13,68]
	Develops moderate to severe respiratory pertussis	0.035	0.021–0.040	[29,30]
	Hospitalization for severe disease	0.027	0.008–0.060	[28,30,73]
	Death	0.0001	0–0.0010	[29,30,75]
	Develops encephalitis/ encephalopathy	0.0005	0.0002–0.0040	[29,30,32]
	Develops chronic neurologic sequelae	0.33	0.25–0.50	[32,33]

^1^ Assumption based on 2 case reports of azithromycin-associated IHPS [36]

^2^ Assumption of range

GI: gastrointestinal

IHPS: infantile hypertrophic pyloric stenosis

PEP: post-exposure prophylaxis

### Intervention

An RCT of erythromycin prophylaxis against pertussis demonstrated 67.5% effectiveness (95% CI 7.6% to 88.7%) in preventing culture-positive pertussis among household contacts [13]. With no subsequent studies re-examining this intervention, this point estimate of effectiveness was extrapolated to azithromycin and clarithromycin based on their comparatively similar efficacy with erythromycin in treatment studies [18,19] which is in keeping with current pertussis PEP guidelines [15,16]. PEP was assumed to have been given within 21 days of onset of cough in the index case and before a secondary case had occurred [28]. Clarithromycin has not been studied for those under 1 month of age and so was not included as an option for the infant group [19].

All contacts who receive an antibiotic have a likelihood of developing a gastrointestinal adverse event. These probabilities were derived from case series and clinical trials involving erythromycin, clarithromycin and/or azithromycin. Neonates may be more than 10 times at risk for infantile hypertrophic pyloric stenosis (IHPS) in the month following orally administered erythromycin, compared to unexposed neonates, with a peak incidence of 1.28% in under 3 month olds [34,35]. As the risk of IHPS with erythromycin has had an impact on recommendations for infant PEP, this was explicitly examined in the model. There were 2 case reports of azithromycin-associated IHPS [36]. With over 4,000 cases noted over a 10-year period in the US [29] and azithromycin having been adopted as the preferred agent for young infants [16], a point estimate of 0.05%, or 2 cases among 4,000 infants treated with azithromycin, was assumed.

If no PEP was received, or no gastrointestinal event occurred on chemoprophylaxis, then erythromycin was used for treatment of pertussis as the least expensive option [37]. If IHPS occurred during erythromycin PEP, infants who subsequently developed pertussis received azithromycin for treatment [16]. However, if IHPS followed azithromycin PEP, then infants with subsequent pertussis were treated with erythromycin. As azithromycin and clarithromycin have fewer side effects than erythromycin, child and adult contacts that developed pertussis after experiencing PEP-related side effects were treated with either of these agents.

#### Health outcomes

QALYs combine quality of life and duration of life, or life years, into a single measure. Health states were valued with preferences (Table 2) drawn from individuals with pertussis [38]. In this study, time trade-off and contingent valuation methods were used to determine utilities of short-term health states among adult patients and parents of adolescent patients diagnosed with pertussis [38]. Infant utilities were derived from the adult respondents, who were presented with the scenario of a 1-month old who developed pertussis, and asked to value the prevention of short-term health states, that is respiratory or neurologic complications lasting 8 weeks’ duration, as compared to long-term health states, which reflect neurologic sequelae [38].

**Table 2:** Utility values and duration of relevant health states.

**Health State**	**Utility value for base case (SD)**	**Days in first year**	**Reference (utility; duration)**
INFANT			
Mild illness, outpatient	0.67 (0.30)	76	[40,41]
Hospitalization	0.58 (0.37)	8	[38,39]
Respiratory complications	0.58 (0.37)	8	[38]
Encephalitis without sequelae	0.51 (0.38)	14	[38]
Chronic neurologic sequelae^1^	0.77 (0.25)^2^	365	[38]
IHPS	0.51 (0.38)^3^	6	Assumption; [34,43]
CHILD			
Mild cough, outpatient	0.85 (0.26)^4^	76	[38,41]
Moderate-severe cough, outpatient	0.81 (0.30)	76	[38,41]
Hospitalization with recovery	0.67 (0.33)	3	[38,42]
Encephalitis without sequelae	0.51 (0.38)	14	[38]; Assumption
Chronic neurologic sequelae	0.77 (0.25)	365	[38]; Assumption
GI adverse event	0.70 (0.15)^5^	7	[84]; Assumption
ADULT			
Mild cough	0.85 (0.26)	87	[38,40]
Moderate cough	0.81 (0.30)	87	[38,40]
Hospitalization for respiratory complications	0.62 (0.40)	3	[38,42]
Encephalitis without sequelae	0.51 (0.38)	14	[38]; Assumption
Chronic neurologic sequelae	0.77 (0.25)	365	[38]; Assumption
GI adverse event	0.70 (0.15)^5^	7	[84]; Assumption

^1^ Utility value and duration in health state assumed to be the same for all age groups

^2^ Interpreted as willing to give up 84 days of life to prevent 1 year of neurologic sequelae

^3^ Assumed to be same as for encephalitis

^4^ Interpreted as willing to give up 8 days of life to prevent 8 weeks of mild cough that does not require hospitalization

^5^ Standard error

GI: gastrointestinal

IHPS: infantile hypertrophic pyloric stenosis

PEP: post-exposure prophylaxis

The time spent in each health state was derived from studies examining the natural course of pertussis in under-immunized populations [39–43], and was multiplied by the corresponding preference value to calculate the QALYs. The literature search did not identify a study reporting a preference value for IHPS, and so it was assumed to be the same as for encephalitis without sequelae, given the need for hospitalization and short-term disability.

#### Costs

The direct medical costs were expressed in 2012 Canadian dollars, and were associated with exposure to and treatment of pertussis. These costs include contact tracing by a public health nurse to review the need for PEP within households, medications, GP visits, diagnostic testing, hospitalization and long-term medical costs associated with neurologic sequelae. While there is no universal drug coverage in Ontario, medication costs were included in this analysis as Canadian public health policy makers have recommended that prophylaxis, where appropriate, should be supplied by public health [15]. Cost estimates are summarized in Table 3. Costs of health care visits and procedures were obtained from the Ontario Health Insurance Plan Schedule of Benefits [44], and prices for generic formulations from the Ontario Drug Benefit Formulary [37,45].

**Table 3:** Estimated direct medical costs per contact. Costs in Canadian dollars, 2012 valuation.

**Parameter**		**Base case value**	**Standard Error**	**Reference**
GENERAL				
Contact tracing (public health)		37.74	-	Assumption^1^
Visit to GP		33.70	-	[44]
Chest X-ray (professional and technical cost)		32.65	-	[44]
No pertussis		0	-	Assumption
MEDICATIONS				
Erythromycin^2^	Infant	2.00	-	[45]
	Child	13.58	-	[37]
	Adult	10.24	-	[37]
Azithromycin^3^	Infant	8.90	-	[37]
	Child	42.87	-	[37]
	Adult	7.84	-	[37]
Clarithromycin^4^	Child	8.16	-	[37]
	Adult	11.54	-	[37]
PERTUSSIS				
GI symptoms in child or adult contact		33.70^5^		[44]
Outpatient with mild illness		GP + treatment		Assumption
Outpatient with moderate-severe illness		2 GP visits + 1 chest x-ray + treatment		Assumption
Hospitalization^6^		12,160	5,689	[39]
IHPS		10,340	550	[43]
COMPLICATIONS				
Encephalitis		27,643	8370	[39]
Chronic neurologic sequelae^7^		103,652	148,867	[50]

^1^ Public health nurse with 4 years of seniority paid an hourly rate of CAD 37.74 (personal communication, Public Health Ontario, July 2012)

^2^ erythromycin at 40 mg/kg/day divided thrice daily for 7 days, maximum 200 mg/day; dispensed at $0.0713 per 50mg/mL and $0.1828 per 250 mg tablet; cost is for 5-kg infant, 34-kg child, and adult contact

^3^ azithromycin at 10 mg/kg on Day 1 followed by 5 mg/kg once daily for 4 days, maximum 500 mg on Day 1 followed by 250 mg for 4 days; dispensed at $5.9347 per 100mg/5mL and $1.3070 per 250mg tablet

^4^ clarithromycin at 15 mg/kg/day divided twice daily, maximum 1000g/day, dispensed for a child at $0.5712 per 250mg/5mL; and an adult at $0.4122 per 250mg tablet

^5^ Cost of 1 GP visit for clinical assessment and antibiotic prescription

^6^ Hospitalization includes the infant with an uncomplicated admission; infant with respiratory complications; child and adult hospitalizations; death in all groups

^7^ Mean cost and standard deviation over 2-year period; includes initial hospitalization

GI: gastrointestinal

GP: general practitioner

IHPS: infantile hypertrophic pyloric stenosis

PEP: post-exposure prophylaxis

The doses and dispensing costs for each macrolide are outlined in Table 4. Given the infant contact is at highest risk for complications with pertussis in the first 4 months of life [9,46], the model assumed a 6-week old infant whose weight was at the 50^th^ percentile-for-age, or 5 kg, as per the CDC Growth Charts for the United States [47]. Thus, the cost of erythromycin was $2 and azithromycin $8.90. Similarly, a child was assumed to have completed the primary immunization series and prefer taking liquid preparations of antibiotics, so that a 10-year old child whose weight was at the 50^th^ percentile-for-age at 34 kg had erythromycin at a cost of $13.58, azithromycin at $42.87 and clarithromycin at $8.16. An adult was assumed to be 30 years old and weigh 70 kg, so that erythromycin cost $10.24, azithromycin $7.84 and clarithromycin $11.54, respectively. The duration of prophylaxis and treatment of pertussis was as per Health Canada guidelines [15].

**Table 4:** Macrolide strategies for pertussis PEP among household contacts.

**Strategy**	**Dose (oral)**	**Cost**	**Duration (days)**
Erythromycin	40 mg/kg/day divided three times daily (maximum 2,000 mg/day)	$0.0713 per 50 mg/mL and $0.1828 per 250 mg tablet	7
Azithromycin	10 mg/kg (maximum 500 mg) on day 1 followed by 5 mg/kg (maximum 250 mg) once daily	$5.9347 per 100mg/5mL and $1.3070 per 250mg tablet	5
Clarithromycin^1^	15 mg/kg/day divided twice daily (maximum 1,000 mg/day)	$0.5712 per 250mg/5mL and $0.4122 per 250mg tablet	7

^1^ Not included as strategy among infants

Medications, GP visits and diagnostic testing are set by the Ministry for all practitioners and pharmacies. Older children and adults with PEP-related gastrointestinal symptoms were assumed to visit their GP to discuss discontinuation of therapy. Acquiring the disease incurred further costs.

Hospitalization costs for pertussis and encephalitis were drawn from the Ontario Case Costing Initiative (OCCI), which collects case costing data for acute inpatient, day surgery and ambulatory care cases, complex continuing care and community care cases [39]. The mean cost per hospitalized case includes costs associated with ward and intensive care, and diagnostic and therapeutic resources. During the fiscal year 2010–2011, there were 10 pertussis-related hospitalizations and fewer than 5 associated encephalitis cases. Fifty-one cases of encephalitis associated with other infectious etiologies were reported to the OCCI and those costs were reviewed. Physician costs are not part of the OCCI, but an estimate of 5% of hospitalization costs was used based on previous studies of influenza analyzing health administrative data (expert opinion, based on [48,49]).

There are no data on long-term outcomes of patients with pertussis encephalitis. Direct medical costs associated with the long-term neurologic sequelae of pertussis encephalitis were extrapolated from a review of direct medical costs for children with complex medical conditions in Ontario. Excluding initial hospitalization costs, the average costs of care for a child with neurological impairment and technology assistance (including cerebrospinal fluid ventricular shunt, gastrostomy, or tracheostomy) was $34,574 (interquartile range: $10,178-$97,063) over a 2-year period [50]. This population was comparable with respect to burden of disease and resource utilization patterns.

### Analysis

Base case analysis was conducted for a previously healthy 6-week old infant at 5 kg; 10-year old child at 34 kg; and 30-year old adult [51,52]. Deterministic sensitivity analysis was performed to explore uncertainty in the point estimates, and a threshold analysis was performed for PEP effectiveness. The ranges examined in the deterministic sensitivity analysis were derived either from the confidence intervals of the point estimate used for the base case; or, if not available, minimum and maximum values in the model were derived from point estimates in other observational studies (Table 1). Probabilistic sensitivity analysis was performed to address uncertainty involving parameters with an underlying probability distribution, varying all selected variables simultaneously [53]. In this assessment, beta distributions for probabilities and utilities and gamma distributions for costs were derived, based on mean and standard error [54]. The model was run 10,000 times so that each event of interest may occur at least once in the simulation. Results were summarized as cost-effectiveness acceptability curves.

## Results

From the model, PEP with azithromycin prevents 439 secondary cases per 1,000 infant contacts, and 41 secondary cases per 1,000 child and adult contacts. The results for the base case analysis are shown in Table 5. A previously-healthy 5-kg infant contact who did not receive PEP experienced 19.22 QALYs, whereas a macrolide resulted in at least 0.45 more expected QALYs, or 165 quality-adjusted life days. Between the macrolides, azithromycin dominated erythromycin with lower expected costs ($1,976 versus $2,096) and outcomes (19.6661 versus 19.6660 QALYs). These findings were sensitive to costs of hospitalization, costs of encephalitis and probability of hospitalization, death, PEP effectiveness and acquiring disease following exposure, and not sensitive to the probability of complications, death, annual costs of neurologic sequelae, probability of an IHPS event with either macrolide, and changes in utility on PEP or with any pertussis health state. However, in all scenarios in univariate and probabilistic sensitivity analyses, azithromycin dominated erythromycin and no PEP.

**Table 5:** Costs, effects and cost-effectiveness of prophylaxis with erythromycin, azithromycin or clarithromycin compared with no intervention for household contacts of cases of pertussis, stratified by age group, discounted at 5%.

Age group	Strategy	Average expected cost ($)	Average expected QALY	Difference in cost ($)	Difference in QALY	ICER ($ per QALY)
Infant	azithromycin	1,975.87	19.66612	-	-	
	erythromycin	2,095.70	19.66602	-	-	dominated
	none	5,815.10	19.21593	-	-	dominated
Child	none	35.13	19.37751	-	-	-
	clarithromycin	101.98	19.40780	66.86	0.0303	2,207
	erythromycin	107.85	19.40753	-	-	dominated
	azithromycin	132.04	19.40957	30.06	0.0018	16,963
Adult	none	14.22	18.72856	-	-	-
	azithromycin	90.25	18.76004	76.02	0.0315	2,415
	erythromycin	97.74	18.75800	-	-	dominated
	clarithromycin	98.37	18.75827	-	-	dominated

**Fig 2:**
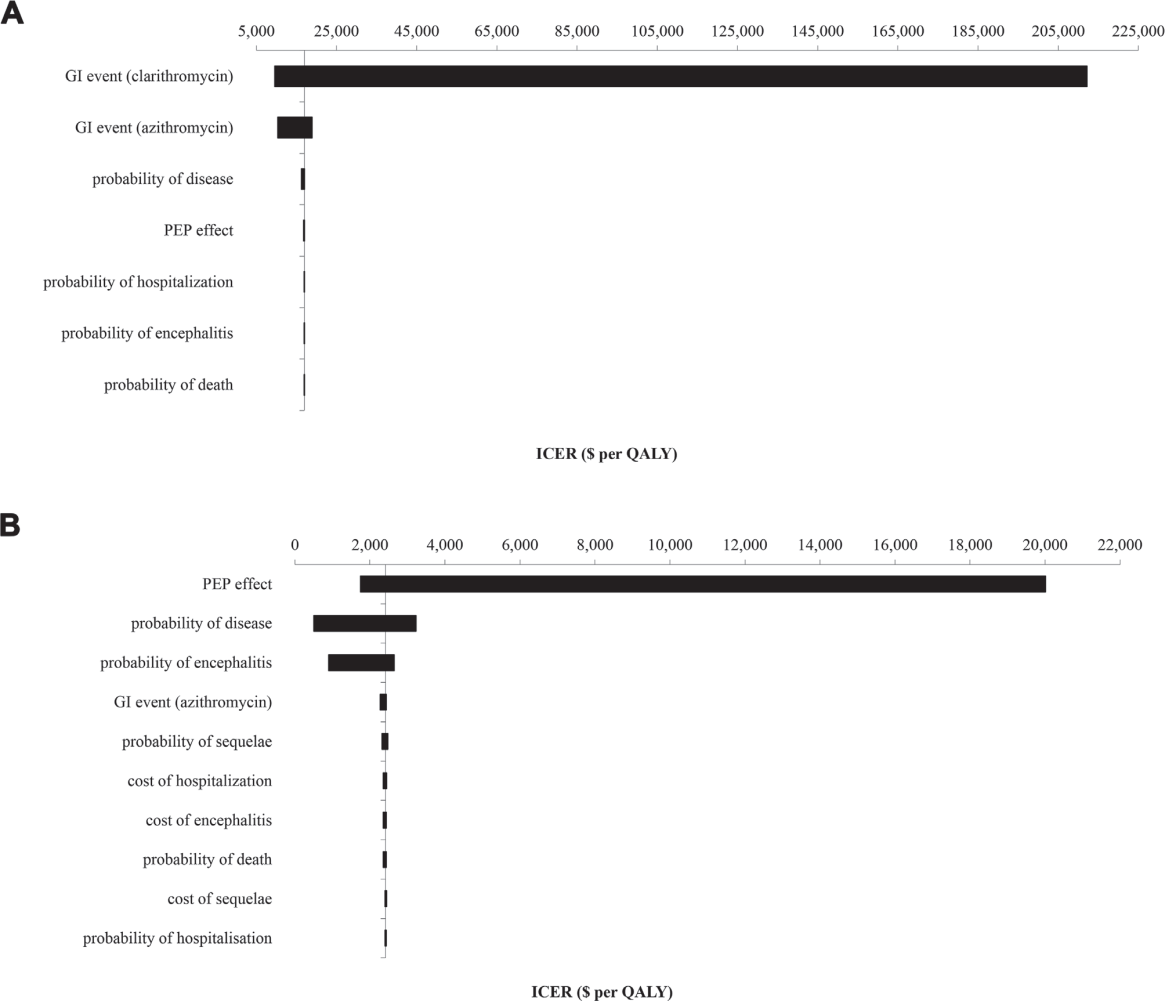
Tornado diagram of the univariate sensitivity analysis for the azithromycin PEP strategy for child (A) and adult (B) contacts. Azithromycin remained the dominant strategy among infants. The bars represent the variation in cost-effectiveness ratios from the base case scenario in response to sequential changes in model parameters, with the vertical axis reflecting the base case ICER. The maximal and minimal values were tested according to ranges outlined in Table 1. (A) Axis at $16,963 per QALY. (C) Axis at $2,415 per QALY.

**Fig 3:**
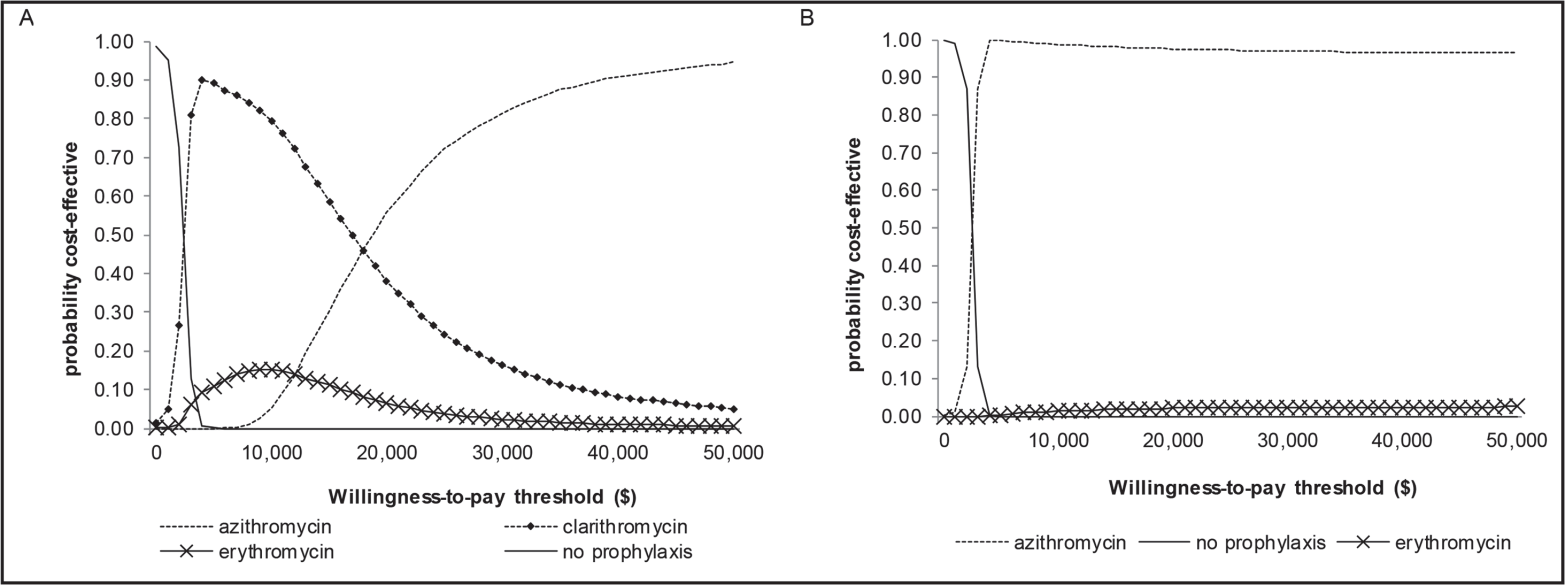
Cost-effectiveness acceptability curves for child (A) and adult (B) contacts. These curves reflect the proportion of times each intervention is likely to be cost-effective for a given cost-effectiveness threshold, up to $50,000 per additional QALY. Clarithromycin post-exposure prophylaxis was never a preferred strategy for adult contacts, and so does not feature for clarity.

The lifetime expected health outcomes for a previously healthy 10-year-old contact were 19.38 QALYs without intervention and increased by 12 days (0.032 QALYs) with azithromycin PEP, with a $97 cost increase for an ICER of $16,963 per QALY when compared with clarithromycin. While clarithromycin was less expensive than azithromycin, there were fewer QALYs gained, thus making it the non-preferred option in the base case scenario and sensitivity analyses. In the probabilistic sensitivity analysis, the median ICER of azithromycin compared to clarithromycin was $16,709 per QALY (mean $16,966 per QALY, 95% CI: $16,900-$16,972 per QALY), and was sensitive to the probability of a GI adverse event with clarithromycin and azithromycin, the probability of acquiring disease, and PEP effectiveness, but not sensitive to the complications and costs of pertussis, including probability and cost of hospitalization, the probability and costs of encephalitis and its sequelae, probability of death, and changes in utility on PEP or with any pertussis health state (Fig. 2A). No PEP had a 95% probability of being cost-effective when the willingness-to-pay threshold was below $2,000 per QALY, whereas azithromycin had a 95% probability of being cost-effective at the threshold of $50,000 per QALY (Fig. 3A).

Among adult contacts, the quality-adjusted life expectancy was 18.73 years without PEP. Azithromycin was the dominant strategy compared to the other macrolides, with more QALYs and lower costs. Compared to no intervention, which was the least costly option, PEP with azithromycin resulted in an additional 0.031 QALYs, or 11 quality-adjusted life days, and $76 in incremental costs, for an ICER of $2,415 per QALY. The median ICER was $2,574 per QALY (mean $2,426 per QALY, 95% CI $2,425-$2,426 per QALY) and was sensitive to PEP effectiveness, probability of acquiring disease and probability of encephalitis, and not sensitive to the probability of an adverse GI event with azithromycin, sequelae and death, costs of hospitalization and encephalitis, and changes in utility on PEP or with any pertussis health state; however, azithromycin remained the preferred strategy (Fig. 2B). Among adult contacts, azithromycin was the preferred strategy, with a 95% probability of being cost-effective at $4,000 per QALY (Fig. 3B).

A threshold analysis for the effectiveness of PEP in preventing a secondary case of pertussis is shown in Table 6. Among infants, when the PEP effect was 0.2% or greater, azithromycin offered the highest net benefit of all strategies. Among children and adults, this threshold probability was 10% and 9%, respectively. Below these values, none of the macrolides offered a higher net benefit than no post-exposure prophylaxis.

**Table 6:** Threshold analysis of post-exposure prophylaxis effectiveness.

	PEP effect									
Contact Group	0	0.001	0.002	0.005	0.01	0.08	0.09	0.1	0.9	1
Infant	no	no	A	A	A	A	A	A	A	A
Child	no	no	no	no	no	no	no	A	A	A
Adult	no	no	no	no	no	no	A	A	A	A

“no” indicates no PEP as the preferred option.

“A” indicates azithromycin. When PEP has zero effect, the net benefit of no prophylaxis exceeds that of azithromycin and other macrolides. At or above 10% effectiveness, PEP with azithromycin offers a higher net benefit than no intervention.

## Discussion

A cost-utility analysis was undertaken to examine 4 strategies against pertussis transmission to household contacts. The decision model synthesized evidence from a broad range of published literature and used QALYs as a preference-based outcome measure. Azithromycin offered the most QALYs in all age groups. Among infants, PEP with azithromycin had the lowest expected cost and most QALYs. This strategy was cost-effective among children and adult contacts at $16,963 and $2,415 per QALY gained, respectively, compared to no PEP, which was well under the cost-effectiveness threshold of $50,000 per QALY gained. The findings were robust in sensitivity analyses. In particular, when the probability of disease acquisition and disease severity were minimized (reflecting an immune population), disease-related costs were still significant, especially among infants, and azithromycin remained the preferred strategy.

Pertussis transmission has been mitigated historically through immunization and chemoprophylaxis. While numerous studies have analyzed the impact of varying immunization strategies [32,38,40,55–60], this is the first study to comparatively evaluate the benefits, risks and costs associated with antibiotic PEP options among household contacts.

A recent systematic review found only 2 RCTs related to pertussis PEP [13,61], and insufficient evidence to support erythromycin for all household contacts [62]; although, they may not have been sufficiently powered to detect a significant difference. An argument against PEP is that widespread immunization has been associated with a milder clinical course among secondary cases [41,63–65]. Most chemoprophylaxis studies occurred in the context of an under-immunized adult population [3,5,66,67] and large cohort studies have demonstrated some benefit in offering PEP to household contacts [27,28,68,69]. However, a recent study among a highly-immunized population of health care professionals found no difference in rates of symptomatic pertussis between those who received PEP compared to daily symptom monitoring [64]. The authors questioned whether antibiotic PEP could be eliminated in such populations, given the potential for reduced prescriptions, rates of adverse events, and labor costs associated with contact tracing, evaluation and counseling for PEP. However, all participants had been immunized against pertussis within the previous 2 years, suggesting higher rates of immunity than what may be found in the general community [67]. Thus, the marginal benefits of PEP in this study may have been smaller compared to what may be found in the general population, particularly among infants and young children who are at highest risk for severe disease [29,42,66].

The analysis has several limitations. The model does not account for changes in disease incidence as it is not an infectious disease transmission model; however the probability of acquiring pertussis and the impact of prophylaxis were varied in sensitivity analyses. The healthcare payer perspective includes only direct medical costs related to health care resource utilization, such as medication, laboratory, procedures and personnel, hospitalizations and physician visits [55]. Numerous studies have highlighted the significant indirect medical and non-medical costs of pertussis [32,40,59,60,70–75]. Given that the antibiotic strategies had similar effectiveness, the differences in non-medical costs between each regimen may be minimal. However, the indirect economic benefit of PEP in avoiding pertussis and its complications may have been underestimated, as the model did not account for QALY loss of caregivers looking after contacts with pertussis or with neurologic sequelae following pertussis encephalitis.

The costs incurred during lifeyears gained from the intervention were not included in this model, in keeping with Canadian guidelines on economic evaluations [20]. Whether to include future unrelated healthcare costs, that is costs solely due to changes in survival, has been controversial. Theoretical arguments for [76] and against inclusion [77] of unrelated costs have been advanced and debated [78].To date no consensus has been reached, and to the best of our knowledge, no current guideline on economic evaluation calls for inclusion of unrelated cost as a requirement. The Canadian guideline recommends including unrelated costs only in sensitivity analysis, if at all [20]. While substantial survival benefits may have a major impact on spending, in the case of pertussis, where the highest risk of mortality is among infants and at 0.6%, offering chemoprophylaxis arguably does not have substantial effects on survival. Since these costs were excluded, the cost-effectiveness of the intervention was likely overestimated.

Existing clinical and cost data were applied to each age group used in this model, and so numerous assumptions were made regarding the calculation and application of costs and probabilities where published data were not available. It is arguable that the populations from which secondary attack rates are estimated may differ from the best available data, which is from Quebec during the 1990s. Despite subsequent changes in the immunization schedules, including a booster dose during adolescence and among adults in contact with children, and in vaccine product, from an adsorbed whole cell vaccine to acellular vaccine, there is no prospective surveillance on immunization rates or household secondary attack rates. Moreover, more than half of pertussis cases continue to occur among children and adults reported as being fully immunized for their age [7,9,28,79], and an increasing lack of protection has been found among children within 5 years of completing the childhood immunization series against pertussis [11]. In this economic analysis, to explore the uncertainty in secondary attack rates, the probability of acquiring disease was varied broadly, and did not result in a change in the preferred strategy for contacts in any of the age groups.

The assumption that respiratory and neurologic diseases are mutually exclusive does not necessarily reflect clinical experience; however the associated costs and QALYs of both complications are likely to overlap, and thus be potentially overestimated in this analysis. The utility scores for infants were derived from adolescent and adult respondents, which may have impacted the validity of the QALYs measured. Direct elicitation of preferences can be especially challenging in children due to evolving developmental maturity and their cognitive abilities at various ages to value health; parents may also not reliably report on subjective outcomes of clinical conditions, all of which potentially impact the validity and generalizability of the QALYs measured.[80,81] Furthermore, only respiratory and neurologic complications were included, while other serious health states in the disease spectrum were not assessed, including apneic spells with spontaneous and complete recovery, generalized illness with outpatient care, and long-term neurologic sequelae following encephalitis.

All cases reported to the OCCI were less than 18 years of age, and the costs of encephalitis and long-term neurologic sequelae have been extrapolated from other disease processes. The cost of death was the same as for hospitalization in this model; however end-of-life care is associated with higher costs [46,49]. Uncertainty in the parameter estimates was tested through univariate and probabilistic sensitivity analyses, and did not shift the preferred strategy from the base case scenario.

Alongside policy recommendations for immunization of infants, children, adolescents and adults, there remains an important role for chemoprophylaxis in the prevention of pertussis, especially in outbreaks, when immunization is not immediately protective, and among infants, who are at highest risk for complicated disease. Our analysis suggests azithromycin PEP to be cost-effective when compared with other macrolides and no intervention, protecting household contacts from acquiring infection at acceptable costs.

### Disclaimer

The opinions, results and conclusions reported in this paper are those of the authors. No endorsement by Public Health Ontario is intended or should be inferred.
